# Collision detection method for anchor digging machine water drilling rig

**DOI:** 10.1038/s41598-024-81640-3

**Published:** 2025-01-03

**Authors:** Zhi-xiang Liu, Shu-tong Yan, Kang Zou, Miao Xie

**Affiliations:** 1 Institute of Mineral Resources Exploitation and Utilization Technology and Equipment, Liaoning Technical University, Fuxin, 123000 Liaoning China; 2 School of Mechanical Engineering, Liaoning Technical University, Fuxin, 123000 Liaoning China

**Keywords:** Anchor digging machine, Water drilling rig, Bounding box, Interference analysis, Collision detection, Mechanical engineering, Engineering

## Abstract

Loading water drilling rig on the anchor digging machine can effectively increase the tunneling efficiency. In order to avoid the interference between the water drilling rig and the anchor machine in the working process, it is necessary to calculate the joint variables of the drilling rig accurately. Using the robot kinematics analysis method, the kinematics model of the system is established. At the same time, combined with bounding box collision detection technology, the key parts of the equipment are mathematically modeled. A method for collision detection of onboard water exploration and drainage drilling rig of anchor digging machine is proposed. The simulation results show : The designed collision detection method can be used to guide the attitude adjustment of the equipment during the drilling operation of the equipment, and can accurately analyze the interference characteristics and collision detection of the equipment. It provides a theoretical basis for the automatic excavation of the research object and the automatic control of drilling. The azimuth adjustment range of the water drilling rig for the experiment is : ± 15°, and the pitch angle adjustment range is : + 5° to -10°.

**Supplementary Information:**

The online version contains supplementary material available at 10.1038/s41598-024-81640-3.

With the increase of coal mining depth and mining intensity in China, the safety production of coal mines has been paid more and more attention, among which gas explosion and water damage are more important causes of accidents^1,2^. At present, the general trend of coal mining is to develop in the direction of intelligent and automatic coal mining equipment^3,4^. Coal mine intelligent and automated production can not only further ensure the safety of personnel, but also improve the efficiency of mining and mining accuracy, but also reduce the amount of labor of workers and the requirements of workers ' professional skills. It is an effective method to improve the mining efficiency by loading the water exploration and drainage drilling rig in the anchor machine and realizing its automatic control. How to ensure the accurate automatic control of airborne drilling rig is a key problem to be studied. Although experts and scholars have done a lot of research on drilling technology and water exploration and drainage drilling rig in water disaster prevention and control instant noodles, there are few studies on the interference of loading water exploration and drainage drilling rig on anchor digging machine. In order to realize the automatic control of the drilling rig, it is necessary to analyze the interference characteristics of the two devices and put forward the corresponding collision detection method to avoid the collision problem between the water exploration drilling rig and the anchor machine.

At present, experts and scholars have made a lot of research on drilling technology and water drilling rig in water disaster prevention and roadway excavation technology. Reference^5^ designed a set of intelligent electric control system for mining horizontal directional drilling rig aiming at the problems of low intelligence, single function and poor safety of the electric control system of domestic horizontal directional drilling rig. It provides a design idea for automatic control of mine drilling rig. Reference^6^ discussed the motion law of the cutting head through the kinematic analysis of the roadheader, and then studied the contour forming of the roadway roof. In reference^7^, the onboard drilling manipulator of roadheader is designed and studied, and its structure, performance and stability of the whole machine are studied in depth by combining with kinematics analysis.

Although the research on the whole machine and related equipment of the roadheader is a hot spot at present, most of them focus on the design of the electronic control system, the mechanical properties of the mechanical structure, and do not involve the accurate control of effectively improving the mining efficiency. How to achieve precise control of equipment while avoiding collisions between devices is a problem that needs to be studied. Therefore, the research aims to design a collision detection system to solve this problem.The research on collision detection methods by scholars at home and abroad includes. Reference^8^ proposed a collision detection, recognition and response method based on dynamic model. The dynamic model of the robot is identified. A dynamic threshold collision detection method is designed. A collision position and direction estimation method is designed, and a robot collision response strategy is proposed. Reference^9^ proposed a research strategy of using friction as an interference term in the motion of the manipulator for collision detection. The external torque observer is used to measure the friction force, and the model parameters are identified by genetic algorithm. The accuracy of the constructed disturbance model and the performance of the proposed collision detection method are verified by experimental research. Reference^10^ proposed Voronoi-Clip or V-Clip collision detection algorithms for polyhedral objects with specified boundaries. The theoretical principle of V-Clip is introduced, and the pseudo-code description of the algorithm is given. V-Clip, Lin-Canny and Enhanced GJK, a simplex-based algorithm widely used in the same application, are compared. Reference^11^ proposed a collision detection framework based on deep learning method ( CollisionNet ). A deep neural network model is designed to learn the robot collision signal and identify any collision. The performance of the proposed framework is verified by various experiments. In Reference^12^, in order to solve the problem that the drilling and anchoring robot cannot monitor the movement of assistants in real time in the fast tunneling system, which may lead to unexpected collisions between the drilling rig and the assistants, a method of human–machine safety collision avoidance for the drilling and anchoring robot was proposed from the perspective of the control system of the drilling and anchoring robot. In Reference^13^, a simulation system based on virtual reality technology was developed to solve the problem of insufficient interaction in the design process of shield machine. In the virtual system, the realization of various movements of the mechanism and various ways of collision detection are mainly considered. In reference^14^, in order to avoid dynamic obstacles in time when the manipulator performs manufacturing tasks, based on the real-time obtained nearest distance between convex hulls of arbitrary shape dynamic obstacles, a new method based on distance calculation and discrete detection is proposed. Reference^15^ proposed a real-time collision detection algorithm for marine robot operation. The proposed collision detection mechanism was developed and integrated into the commercial ROV manipulator control system, and was successfully evaluated in simulation and experimental devices using a real industry-standard underwater manipulator. Reference^16^ proposed a collision detection system based on nonlinear adaptive impedance control law. The collision between the manipulator and the environment is detected without the use of external sensors. Adaptive impedance control is used to estimate the dynamic parameters of the manipulator, allowing the manipulator to interact with the environment. Reference^17^ Combining the advantages of AABB and OBB, a hybrid bounding box collision detection algorithm: AABB-OBB is proposed. It applies to solid objects. A simpler bounding volume tree is constructed, and an improved data structure is applied to reduce the storage space. In the process of traversing AABB-directed bounding box trees, a single traversal method is used to improve the efficiency of collision detection, especially when the depth of trees varies greatly, the effect is more obvious. In References^18,19^ combined with digital twin technology, constructed a virtual simulation system through software such as Unity 3D, and applied bounding box technology to analyze the collision of research objects. Reference^20^ introduced the collision detection algorithm for convex polyhedron, which laid the foundation for future generations to study collision detection. The research methods and research ideas used in the above literature provide some help and inspiration for this study.

Although many scholars have invested a lot of energy in these aspects, in the research of some robot collision detection methods, it is not suitable for the influence of narrow space in coal mines, dust and humidity and other environmental particularities on detection accuracy and reliability. Therefore, a complete research system on collision detection and safety control of intelligent mining robots in coal mines needs to be improved. At the same time, the systematic research on the cooperative work and safety management between robots and other equipment and environment in the process of intelligent mining in coal mines, the detailed analysis and verification of practical application cases also need to be improved.

Inspired by the above problems, it is necessary to analyze the interference characteristics of the two devices and propose corresponding collision detection methods. And on this basis, a collision detection system is designed.The contributions are highlighted as follows:

1. Based on the kinematic analysis of the equipment, the kinematic model of the equipment is established. At the same time, the key parts of the equipment are mathematically modeled with the bounding box collision detection technology, and the corresponding collision detection method is designed. Different from the literature on collision detection of manipulators, shield machines, etc., this paper specifically studies the water drilling rig of anchor digging machine.
2. The feasibility and accuracy of the method are verified by simulation analysis. Based on the collision detection method, the corresponding collision detection system is designed.
3. Different from the literature of simple theoretical analysis, this paper comprehensively verifies the accuracy and reliability of the detection method by conducting experiments on the experimental platform of the coal mine underground environment. It provides a strong basis for the practical application of the detection method.

As a consequence, the rest of this article is organized as follows. In the second section, the preliminary knowledge is given, and the working conditions of the two equipment are briefly introduced. In the third section, the corresponding collision detection method is designed. In the fourth section, the feasibility and accuracy of the method are verified by simulation analysis. The corresponding collision detection system is designed. The fifth section provides an experimental platform for the coal mine underground environment to comprehensively verify the accuracy and reliability of the detection method. Then, the conclusion is drawn in the sixth part.

## Drilling rig workflow and working condition analysis

**Fig. 1:**
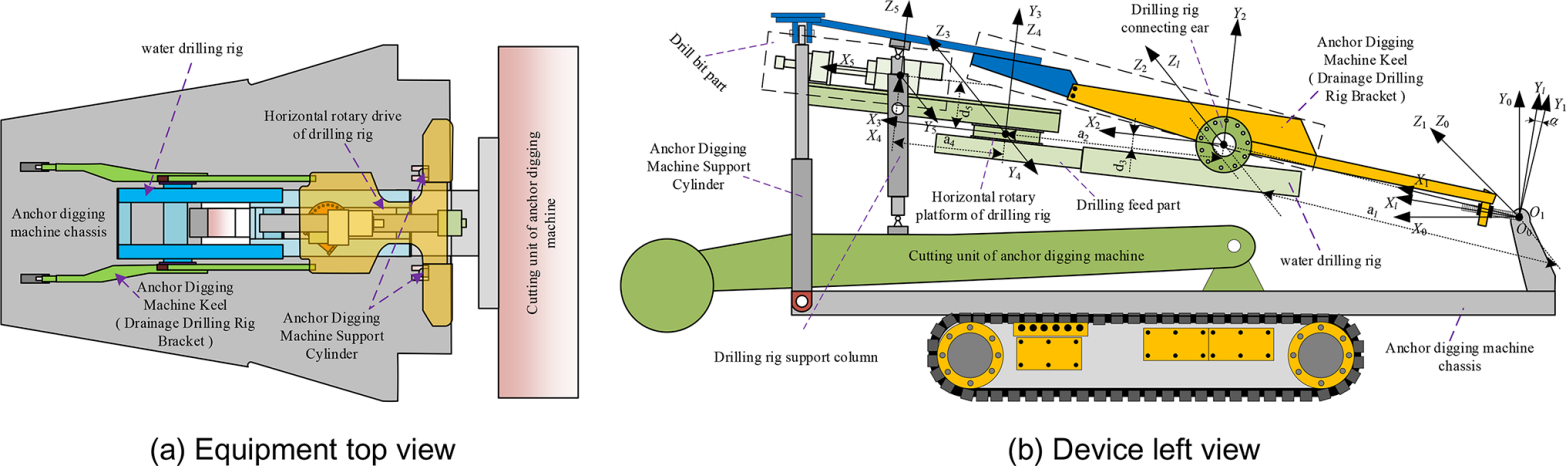
Equipment diagram and D-H coordinate system.

**Fig. 2:**
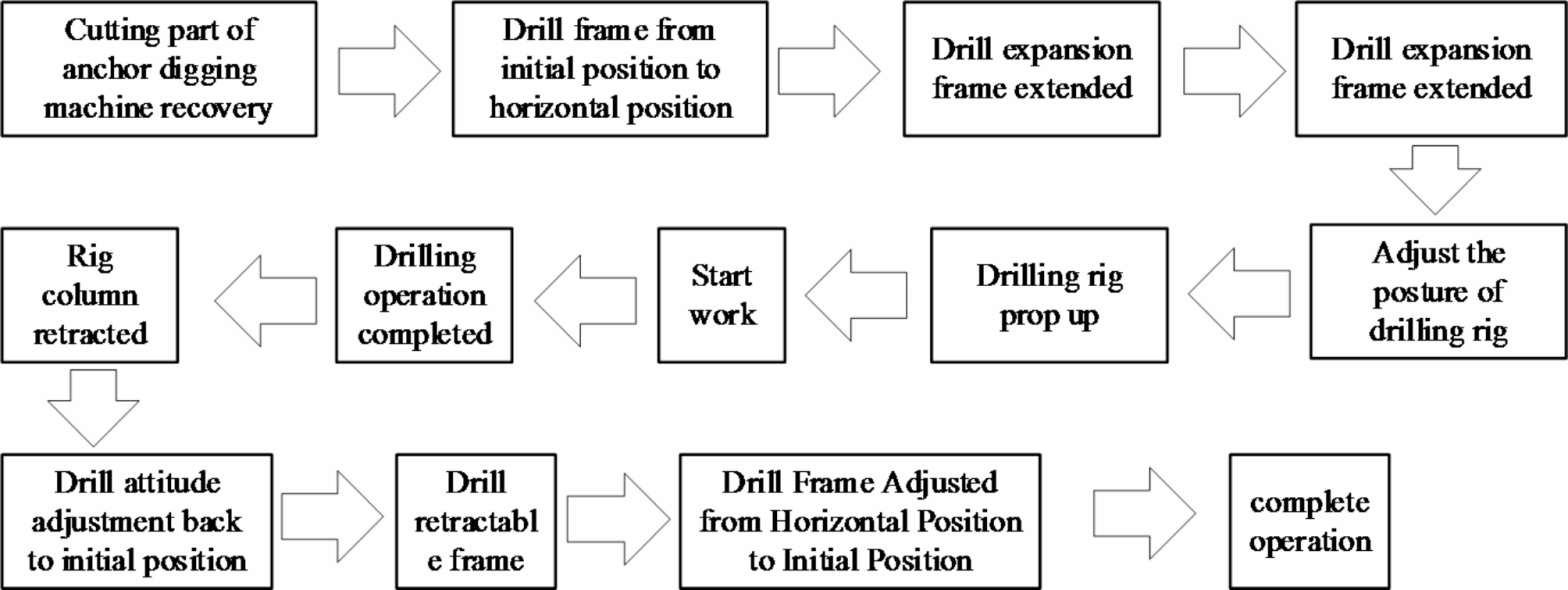
Working process of water drilling rig.

In order to effectively prevent floods, in the underground excavation, the anchor digging machine is required to use the water drilling rig to drill forward for a distance in advance for each distance of excavation. Therefore, loading the water drilling rig on the anchor digging machine can effectively improve the tunneling efficiency of the anchor digging machine. The water drilling rig is installed on the keel of the anchor digging machine and is located at the original ventilation dust removal fan. The installation position on the digging anchor digging machine and the structure of the equipment are shown in Fig. 1 Drilling rig water exploration work flow chart is shown in Fig. 2.

This paper mainly studies the interference characteristics between the drilling rig attitude adjustment and the anchor digging machine in the working process of the rotary machine. This paper mainly studies the interference characteristics between the drilling rig posture adjustment and the anchor digging machine in the drilling rig workflow. According to the working process of the drilling rig, the collision between the drilling rig and the anchor machine mainly occurs between the following parts :

- When the water drilling rig is in the vertical direction of the pitch motion, the drill bit part collides with the upper guard plate of the anchor digging machine keel and the cutting part of the anchor digging machine.
- the collision between the horizontal rotary platform of the water drilling rig and the keel support cylinder of the anchor digging machine when the water exploration and drainage drilling rig is rotated horizontally. Collision between supporting oil cylinder of water drilling rig and keel supporting oil cylinder of anchor digging machine.
- the collision between the support cylinder of the water drilling rig and the keel support cylinder of the anchor digging machine when the water drilling rig is rotated horizontally.

In summary, the collision between the two devices can be summarized as : Collision between drill bit and keel side guard plate, Collision between drill bit and upper keel guard plate, Collision between drill pipe holder of drilling rig and guard plate on keel, Collision between the axis extension line of the rotary center of the drill pipe holder and the cutting part of the anchor digging machine ( collision between the drill pipe and the cutting part of the anchor digging machine ) and Collision between drilling rig support cylinder and keel support cylinder.

## System mathematical model

In Sect. 1, the collision relationship between the two devices is preliminarily determined by analyzing the working conditions of the equipment. For further research, this paper mathematically expresses the collision between two devices through the following ideas:

- Establish a D-H coordinate system between the two devices, solve the kinematics model of the system, and express the motion of the two devices.
- Based on the position relationship between the drilling rig and the anchor digging machine before the attitude adjustment of the drilling rig, the corresponding bounding box or boundary is established based on the coordinate system of each component. The two keel side guard plates and the keel upper guard plate are simplified into three planes, and the corresponding plane mathematical expressions are given. The drill bit is simplified into a cube and mathematically expressed. The drill pipe holder of the drilling rig is simplified as a hollow cylinder and given a mathematical expression. The drilling rig support cylinder, the keel support cylinder and the cutting part of the anchor digging machine are simplified as solid cylinders, and the three parts are given corresponding mathematical expressions.
- The mathematical expression of the position of the drill bit, the drill holder and the support cylinder of the drill after the attitude adjustment is obtained through the pose matrix.
- Collision detection of the system to obtain a collision mathematical model.

### System kinematics model

Define the system base coordinate system ( world coordinate system ) is* O*_0_*X*_0_*Y*_0_*Z*_0_, anchor digging machine and water drilling rig each mechanism coordinate system is *O*_*i*_*X*_*i*_*Y*_*i*_*Z*_*i*_ ( where : *i* = 1 represents the keel coordinate system of the anchor digging machine ; *i* = 2 represents the expansion frame coordinate system of the water drilling rig ; *i* = 3 represents the coordinate system of the drilling rig mobile platform ; *i* = 4 represents the coordinate system of the rotary platform of the drilling rig ; *i* = 5 represents the drill bit feed coordinate system.). According to the positional relationship and motion relationship between the two devices, the D-H coordinate system of the system is established as shown in Fig. 2 (b). According to the D-H coordinate system shown in Fig. 2 (b), the homogeneous transformation matrices describing the keel of the anchor digging machine, the rotary platform of the water drilling rig ( the drill bit guide rail ) and the drill bit in the world coordinate are obtained as shown in Formula ( 1 ), Formula ( 2 ) and Formula ( 3 ) respectively :

$${}_{1}^{0} T = \left[ {\begin{array}{*{20}c} {c_{1} } & { - s_{1} } & {} & {} \\ {s_{1} } & {c_{1} } & {} & {} \\ {} & {} & 1 & {} \\ {} & {} & {} & 1 \\ \end{array} } \right]$$, $${}_{2}^{1} T = \left[ {\begin{array}{*{20}c} {c_{2} } & { - s_{2} } & {} & {a_{1} } \\ {s_{2} } & {c_{2} } & {} & {} \\ {} & {} & 1 & {} \\ {} & {} & {} & 1 \\ \end{array} } \right]$$, $${}_{3}^{2} T = \left[ {\begin{array}{*{20}c} 1 & {} & {} & {a_{2} } \\ {} & 1 & {} & {d_{3} } \\ {} & {} & 1 & {} \\ {} & {} & {} & 1 \\ \end{array} } \right]$$.

$${}_{4}^{3} T = \left[ {\begin{array}{*{20}c} {c_{4} } & { - s_{4} } & {} & {} \\ 0 & 0 & { - 1} & {} \\ {s_{4} } & {c_{4} } & {} & {} \\ {} & {} & {} & 1 \\ \end{array} } \right]$$, $${}_{5}^{4} T = \left[ {\begin{array}{*{20}c} 1 & {} & {} & {a_{4} } \\ {} & 1 & {} & {} \\ {} & {} & 1 & {d_{5} } \\ {} & {} & {} & 1 \\ \end{array} } \right]$$, $${}_{l}^{1} T = \left[ {\begin{array}{*{20}c} {c_{\alpha } } & { - s_{\alpha } } & {} & {} \\ {s_{\alpha } } & {c_{\alpha } } & {} & {} \\ {} & {} & 1 & {} \\ {} & {} & {} & 1 \\ \end{array} } \right]$$

1 $${}_{l}^{0} T = {}_{1}^{0} T{}_{2}^{1} T{}_{3}^{2} T = \left[ {\begin{array}{*{20}c} { c_{\alpha 12} } & { - s_{\alpha 12} } & 0 & {a_{1} c_{1} } \\ {s_{\alpha 12} } & {c_{\alpha 12} } & 0 & {a_{1} s_{1} } \\ 0 & 0 & 1 & 0 \\ 0 & 0 & 0 & 1 \\ \end{array} } \right]$$

2 $${}_{4}^{0} T = {}_{1}^{0} T{}_{2}^{1} T{}_{3}^{2} T{}_{4}^{3} T = \left[ {\begin{array}{*{20}c} {c_{4} c_{12} } & { - s_{4} c_{12} } & { - s_{12} } & {a_{1} c_{1} + a_{2} c_{12} - d_{3} s_{12} } \\ {c_{4} s_{12} } & { - s_{4} s_{12} } & {c_{12} } & {a_{1} s_{1} + a_{2} s_{12} + d_{3} c_{12} } \\ { - s_{4} } & { - c_{4} } & 0 & 0 \\ 0 & 0 & 0 & 1 \\ \end{array} } \right]$$

3 $$\left\{ \begin{gathered} {}_{5}^{0} T = = {}_{1}^{0} T{}_{2}^{1} T{}_{3}^{2} T{}_{4}^{3} T{}_{5}^{4} T = \left[ {\begin{array}{*{20}c} {c_{4} c_{12} } & { - s_{4} c_{12} } & { - s_{12} } & x \\ {c_{4} s_{12} } & { - s_{4} s_{12} } & {c_{12} } & y \\ { - s_{4} } & {c_{4} } & 0 & {a_{4} s_{4} } \\ 0 & 0 & 0 & 1 \\ \end{array} } \right] \hfill \\ \hfill \\ x = a_{1} c_{1} + (a_{2} + a_{4} c_{4} )c_{12} - (d_{3} + d_{5} )s_{12} \hfill \\ y = a_{1} s_{1} + (a_{2} + a_{4} c_{4} )s_{12} + (d_{3} + d_{5} )s_{12} \hfill \\ \end{gathered} \right.$$

In the formula : *a*_1_ is the distance between the axis of the center of the keel installation winch and the rotation axis of the rotary center of the winch connecting the telescopic frame of the drilling rig in the direction of the common vertical line ; *a*_2_ represents the distance from the telescopic frame of the drilling rig to the horizontal direction of the feed platform of the drilling rig ; *a*_4_ represents the horizontal distance from the axis of the rotary platform of the drilling rig to the geometric center of the drilling bit ; *c*_*i*_ and *s*_*i*_ represent *cos*(*θ*_*i*_) and *sin*(*θ*_*i*_) ( *i* = 1,2,3… ) respectively ; *s*_12_, *c*_12_, *s*_*α*12_, *c*_*α*12_ represent *sin* ( *θ*_1_ + *θ*_2_ ), *cos* ( *θ*_1_ + *θ*_2_ ), *sin* ( *α* + *θ*_1_ + *θ*_2_ ), *cos* ( *α* + *θ*_1_ + *θ*_2_ ) respectively ;* d*_2_ indicates the vertical upward distance from the rotation center of the connecting ear of the expansion frame of the drilling rig to the feeding platform of the drilling rig ; *d*_4_ represents the vertical distance from the geometric center of the rotary platform to the geometric center of the drill bit.

### Mathematical expression of key parts of equipment

**Fig. 3:**
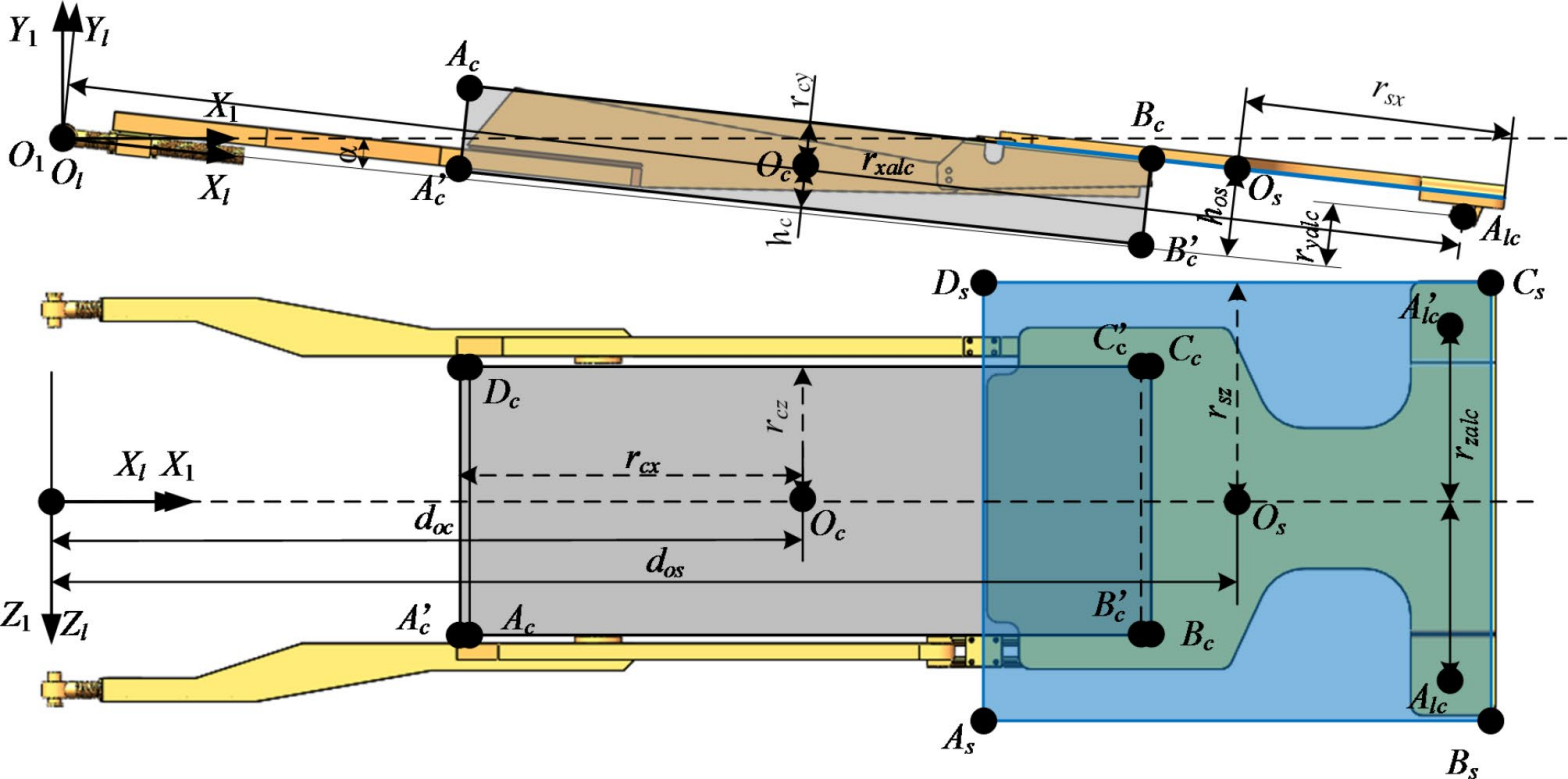
keel bounding box.

Considering that the drilling rig can only move in the space limited by the guard plates on both sides. The AABB bounding box is used to describe the movement range of the drill bit within the boundary defined by the guard plates on both sides of the keel. Considering that the upper guard plate of the keel may collide with the drill bit on one surface, the upper guard plate of the keel is directly expressed as a plane. Further simplification of the keel is shown in Fig. 3.

In Fig. 3 : $A_{c} B_{c} C_{c} D_{c} - A_{c}^{\prime} B_{c}^{\prime} C_{c}^{\prime} D_{c}^{\prime}$ is the bounding box of the space restricted by the guard plates on both sides of the keel, and the surfaces to be detected for collision are bounded planes $A_{c} B_{c} A_{c}^{\prime} B_{c}^{\prime}$ and $C_{c} D_{c} C_{c}^{\prime} D_{c}^{\prime}$;$A_{s} B_{s} C_{s} D_{s}$ is the surface to be detected when the keel upper guard plate collides with the drilling bit ; *O*_*c*_ is the center of the space bounding box limited by the keel side guard plate ; *O*_*s*_ is the center of the bounded plane.

According to the geometric relationship shown in Fig. 3, the expression of the area surrounded by the keel side guard plate is shown in formula (4). The upper guard plate expression of the keel is shown in Eq. (5).

4 $$\delta_{C} \in [O_{C} - r_{C} ,O_{C} + r_{C} ]$$

In the formula : *O*_*C*_ is the geometric center of the enclosure. *r*_*C*_ is the distance from the point *O*_*C*_ to the side of the enclosure in the direction of the coordinate axis, that is, *r*_*C*_ = ( *r*_*cx*_, *r*_*cy*,_ *r*_*cz*_ ).

5 $$\left\{ \begin{gathered} x_{{O_{S} }} - r_{sx} \le x \le x_{{O_{S} }} + r_{sx} \hfill \\ y = y_{{O_{S} }} \hfill \\ z_{{O_{S} }} - r_{sz} \le z \le z_{{O_{S} }} + r_{sz} \hfill \\ \end{gathered} \right.$$

In the formula : *O*_*S*_ is the geometric center of the upper guard plate ; *r*_*sx*_ and *r*_*sz*_ are the distances from *O*_*S*_ to the upper guard plate boundary in the x-axis and y-axis directions.

**Fig. 4:**
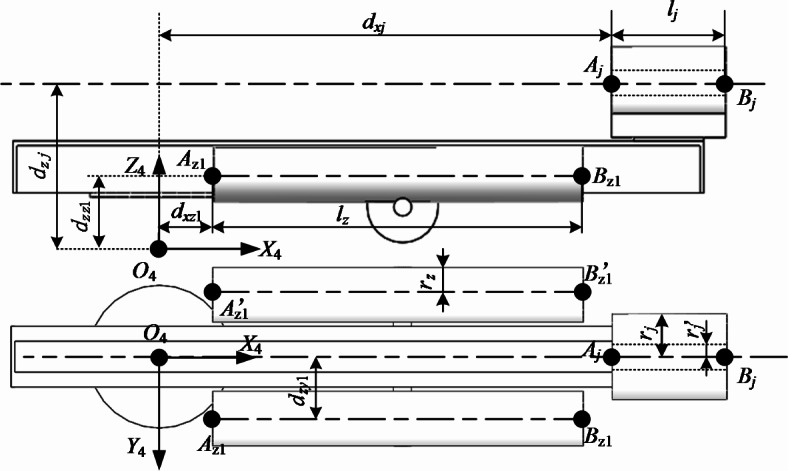
Drilling rig support leg and drill pipe holder.

For the horizontal rotary platform of drilling rig, the corresponding parts are understood as space cylinders and mathematically described. For the drill pipe holder, it is understood as a hollow cylinder. The corresponding structure and size are shown in Fig. 4

In Fig. 4 :* A*_z1_*B*_z1_、${A}_{z1}^{\prime} {B}_{z1}^{\prime}$ is the central axis of the cylinder where the two rig supporting legs are located. *r*_*z*_ is the cylindrical radius of the supporting leg of the drilling rig. *A*_j_*B*_j_ is the hollow cylinder central axis of the drill pipe holder. *R*_*j*_、${R}_{j}^{\prime}$ is the inner and outer cylinder radius of the drill pipe holder.

According to the geometric relationship shown in Fig. 4, the mathematical expression of the supporting leg of the drilling rig is shown as formula (6), and the mathematical expression of the drill pipe gripper is formula (7).

6 $$\left\{ \begin{gathered} \begin{array}{*{20}c} {f(t) = (1 - t)A + tB} & {t \in [0,1]} \\ \end{array} \hfill \\ \begin{array}{*{20}c} {f(t,\theta ) = f(t) + \overrightarrow {{n_{1} }} r\cos \theta + \overrightarrow {{n_{2} }} r\sin \theta } & {\theta \in [0,2\pi ]} \\ \end{array} \hfill \\ \end{gathered} \right.$$

In the formula : $A,B$ represents the two endpoints of the cylinder, namely $A_{z1} ,A_{z1}^{\prime}$ and $B_{z1} ,B_{z1}^{\prime}$. *r* represents the radius of the cylinder $r_{z1} ,r_{z1}^{\prime}$. $\overrightarrow {{n_{1} }} ,\overrightarrow {{n_{2} }}$ is two unit vectors perpendicular to the central axis of the cylinder, and the two vectors are not on the same line.

7 $$\left\{ \begin{gathered} \begin{array}{*{20}c} {f(t) = (1 - t)A + tB} & {0 \le t \le 1} \\ \end{array} \hfill \\ \begin{array}{*{20}c} {f(t,\theta ) = f(t) + \overrightarrow {{n_{1} }} r\cos \theta + \overrightarrow {{n_{2} }} r\sin \theta } & {\theta \in [0,2\pi ]} \\ \end{array} \hfill \\ r_{j}^{\prime} \le r \le r_{j} \hfill \\ \end{gathered} \right.$$

In the formula : $A,B$ represents the two endpoints of the cylinder, namely $A_{j}$ and $B_{j}$. *r* represents the radius of the cylinder $r_{j}$.$\overrightarrow {{n_{1} }} ,\overrightarrow {{n_{2} }}$ is two unit vectors perpendicular to the central axis of the cylinder, and the two vectors are not on the same line.

**Fig. 5:**
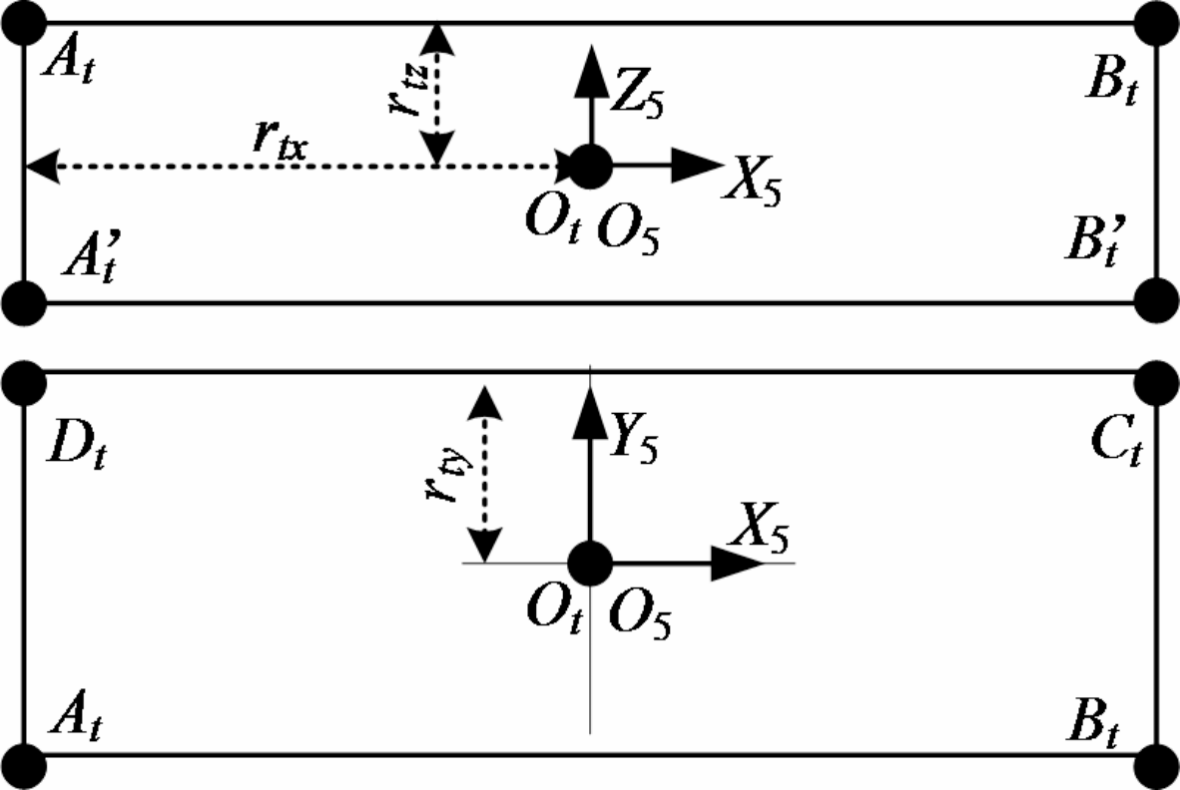
Drilling rig drill simplification.

For the drill bit, the AABB bounding box is used to describe it, and the corresponding simplified model is shown in Fig. 5.

In Fig. 5, *O*_*t*_ is the geometric center of the bounding box. *r*_*tx*_, *r*_*ty*_ and *r*_*tz*_ are the distances from the geometric center of bounding box to each surface of bounding box in the coordinate system *O*_5_*X*_5_*Y*_5_*Z*_5_.

According to the geometric relationship shown in Fig. 5, the mathematical expression of drill bit is :

8 $$\delta_{t} \in [O_{t} - r_{t} ,O_{t} + r_{t} ]$$

In the formula : *O*_*t*_ is the geometric center of the enclosure. *r*_*t*_ is the distance from the point *O*_*t*_ to the side of the enclosure in the direction of the coordinate axis, that is, *r*_*t*_ = ( *r*_*t*x_, *r*_*ty*_, *r*_*tz*_ ).

**Fig. 6:**
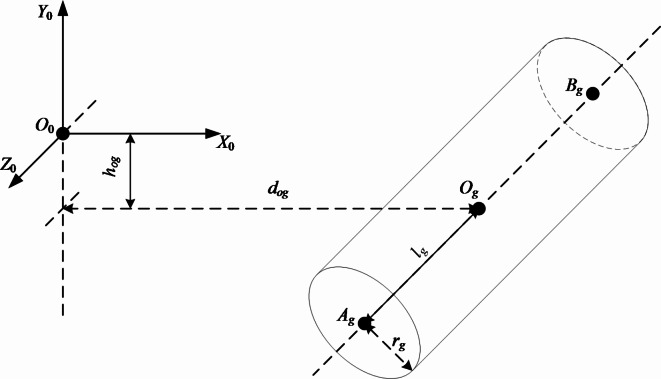
Cutting unit of anchor digging machine.

When the water drilling rig works, the cutting part of the digging anchor digging machine is in the recovery state, and there is no relative motion in the cutting part of the digging anchor digging machine. Therefore, the anchor digging machine is defined as a cylinder in the world coordinate system. The position relationship and structural diagram of the cutting part of the anchor digging machine in the world coordinates are shown in Fig. 6.

In Fig. 6, *A*_*g*_*B*_*g*_ is the central axis of the simplified model of the cutting part of the anchor digging machine, and *O*_*g*_ is its geometric center. According to the geometrical relationship shown in Fig. 6, the mathematical model of the cutting head of the anchor digging machine in the world coordinates can be derived as follows :

9 $$\left\{ \begin{gathered} \begin{array}{*{20}c} {f(t) = (1 - t)A_{g} + tB_{g} } & {t \in [0,1]} \\ \end{array} \hfill \\ \begin{array}{*{20}c} {f(t,\theta ) = f(t) + \overrightarrow {{n_{1} }} r_{g} \cos \theta + \overrightarrow {{n_{2} }} r_{g} \sin \theta } & {\theta \in [0,2\pi ]} \\ \end{array} \hfill \\ \end{gathered} \right.$$

**Fig. 7:**
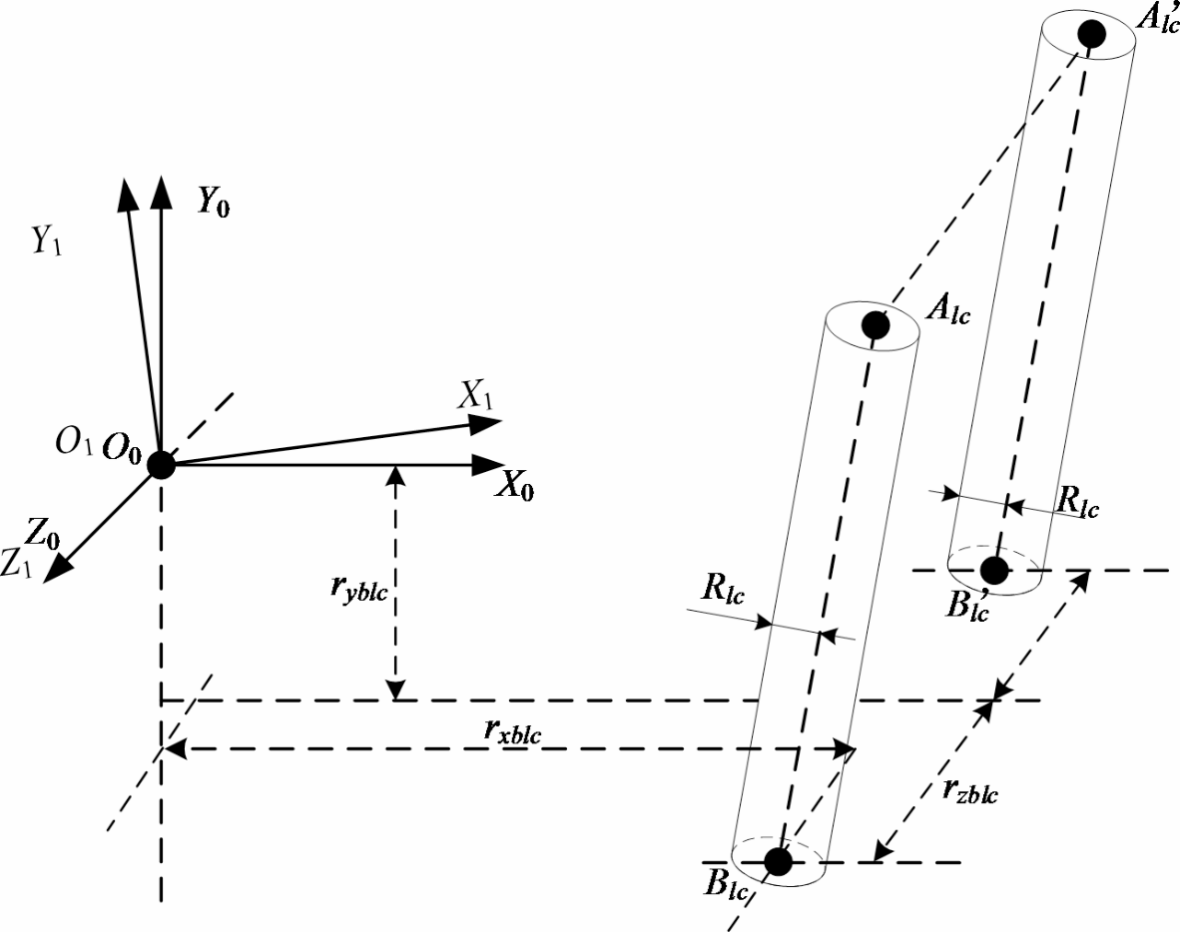
Simplification of keel support cylinder.

Anchor digging machine keel support cylinder, one end hinged on the keel, the other end hinged on the anchor digging machine. Therefore, the two ends of the keel support cylinder are defined with two different coordinate systems, as shown in Fig. 7.

In Fig. 7, the position relation of point $B_{lc} ,B_{lc}^{\prime}$ in the world coordinate is mainly marked. For the position relationship of the point $A_{lc} ,A_{lc}^{\prime}$ on the keel, see Fig. 4, the calculation is transformed to the world coordinate by the pose matrix. The mathematical expression of the keel support cylinder in three-dimensional space is consistent with Eq. (6). When the formula (6) represents the keel support cylinder bounding box,$A,B$ represents $A_{z1} ,A_{z1}^{\prime}$ and $B_{z1} ,B_{z1}^{\prime}$ four points, *r* represents the cylinder radius *R*_*lc*_.

The coordinates of the key points of each key component of the equipment in its own coordinate system are summarized in Table 1.

**Table 1:** Key point coordinates.

Nodal point	Nodal coordinate	Nodal point	Nodal coordinate
*O*_*c*_	(*d*_*oc*_, *h*_*c*_, 0)	$A_{c}$	(*d*_*oc*_*-r*_*cx*_, *r*_*cy*_, *r*_*cz*_)
$B_{c}$	(*d*_*oc*_ + *r*_*cx*_, *r*_*cy*_, *r*_*cz*_)	*C*_*c*_	(*d*_*oc*_ + *r*_*cx*_, *r*_*cy*_,—*r*_*cz*_)
*D*_*c*_	(*d*_*oc*_*-r*_*cx*_, *r*_*cy*_, -*r*_*cz*_)	$A_{c}^{\prime}$	(*d*_*oc*_*-r*_*cx*_, -*r*_*cy*_, *r*_*cz*_)
$B_{c}^{\prime}$	(*d*_*oc*_ + *r*_*cx*_, -*r*_*cy*_, *r*_*cz*_)	$C_{c}^{\prime}$	(*d*_*oc*_ + *r*_*cx*_, -*r*_*cy*_,—*r*_*cz*_)
$D_{c}^{\prime}$	(*d*_*oc*_*-r*_*cx*_, -*r*_*cy*_, -*r*_*cz*_)	*O*_*s*_	(*d*_*os*_, *h*_*os*_, 0)
*A*_*s*_	(*d*_*os*_-*r*_*sx*_, *h*_*os*_, *r*_*sz*_)	*B*_*s*_	(*d*_*os*_ + *r*_*sx*_, *h*_*os*_, *r*_*sz*_)
*C*_*s*_	(*d*_*os*_ + *r*_*sx*_, *h*_*os*_, -*r*_*sz*_)	*D*_*s*_	(*d*_*os*_*-r*_*sx*_, *h*_*os*_, -*r*_*sz*_)
*A*_*z*1_	(*d*_*xz*1_, *d*_*zy*1_, *d*_*zz1*_ )	*B*_*z*1_	(*d*_*xz*1_ + *l*_*z*_, *d*_*zy*1_, *d*_*zj*_ )
$A_{z1}^{\prime}$	(*d*_*zx*1_, -*d*_*zy*1_, *d*_*zz*1_ )	$B_{z1}^{\prime}$	(*d*_*zx*1_ + *l*_*z*_, -*d*_*zy*1_, *d*_*zz*1_ )
*A*_*j*_	(*d*_*xj*_, 0, *d*_*zj*_)	*B*_*j*_	(*d*_*xj*_ + *l*_*j*_, 0, *d*_*zj*_)
*O*_*t*_	(0, 0, 0)	$A_{t}$	(-*r*_*tx*_, -*r*_*ty*_, *r*_*tz*_)
$B_{t}$	(*r*_*tx*_,- *r*_*ty*_, *r*_*tz*_)	$C_{t}$	(*r*_*tx*_, *r*_*ty*_, *r*_*tz*_)
$D_{t}$	(-*r*_*tx*_, *r*_*ty*_, *r*_*tz*_)	$A_{t}^{\prime}$	(-*r*_*tx*_, -*r*_*ty*_, -*r*_*tz*_)
$B_{t}^{\prime}$	(*r*_*tx*_,- *r*_*ty*_, -*r*_*tz*_)	$C_{t}^{\prime}$	(*r*_*tx*_, *r*_*ty*_, -*r*_*tz*_)
$D_{t}^{\prime}$	(-*r*_*tx*_, *r*_*ty*_, -*r*_*tz*_)	*O*_*g*_	(*d*_*og*_, *-h*_*og*_, 0)
*A*_*g*_	(*d*_*og*_, *-h*_*og*_, *l*_*g*_)	*B*_*g*_	(*d*_*og*_, *-h*_*og*_, -*l*_*g*_)
*A*_*lc*_	(*r*_*xalc*_, *r*_*yalc*_, *r*_*zalc*_)	*B*_*lc*_	(*r*_*xalc*_, *r*_*yalc*_, *r*_*zalc*_)
$A_{lc}^{\prime}$	(*r*_*xblc*_, *r*_*yblc*_, -*r*_*zalc*_)	$B_{lc}^{\prime}$	(*r*_*xblc*_, -*r*_*yblc*_, -*r*_*zalc*_)

## Collision detection method

**Fig. 8:**
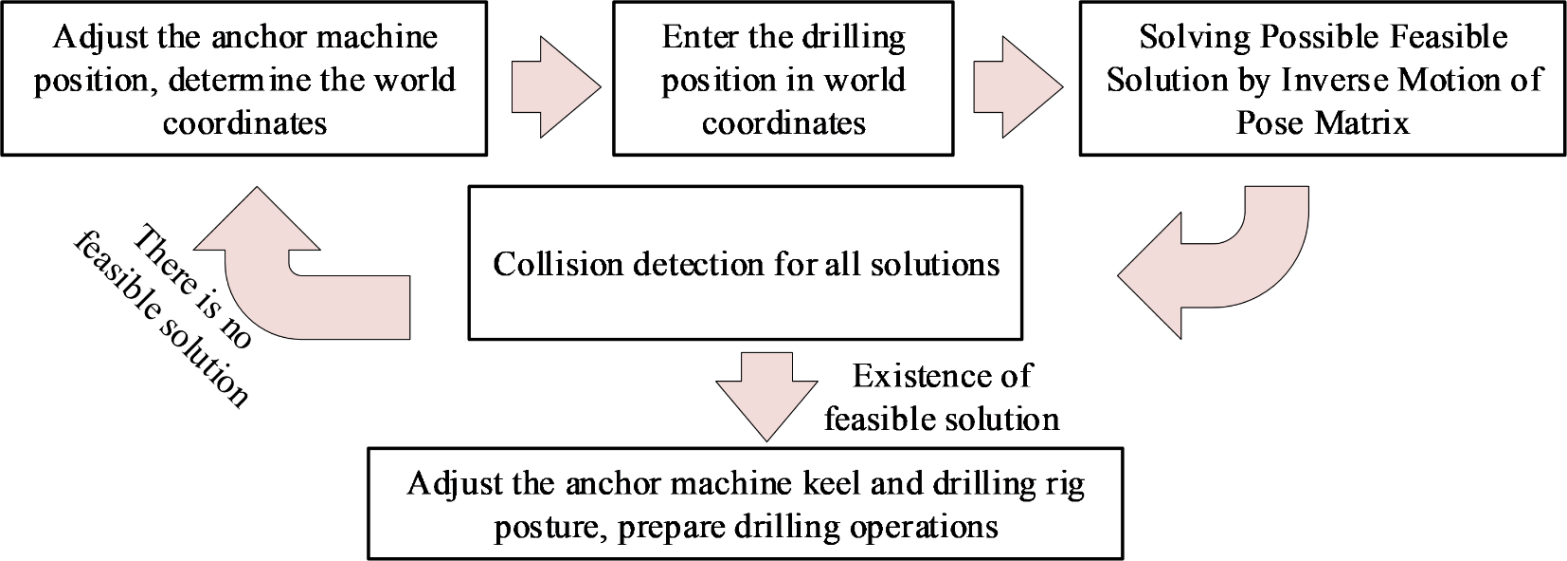
Collision detection process.

When the operation of the anchor digging machine is completed and the water drilling rig is drilling, the collision detection process between the anchor digging machine and the water drilling rig is shown in Fig. 8.

Combined with the actual working conditions, it can be seen that the collision form between the keel side guard plate of the anchor digging machine and the drill bit of the water drilling rig is described mathematically as follows : The collision between the edge $A_{t} A_{t}^{\prime}$ and edge $D_{t} D_{t}^{\prime}$ of the bit bounding box $A_{t} B_{t} C_{t} D_{t} - A_{t}^{\prime} B_{t}^{\prime} C_{t}^{\prime} D_{t}^{\prime}$ and the side $A_{c} B_{c} A_{c}^{\prime} B_{c}^{\prime}$ and $C_{c} D_{c} C_{c}^{\prime} D_{c}^{\prime}$ of the keel bounding box. There is no collision between the two parts if and only if the two straight lines are inside the keel bounding box. Therefore, the method and steps of collision detection between keel side plate and drill bit are :

- The coordinates of each poin $A_{t} ,A_{t}^{\prime} ,D_{t} ,D_{t}^{\prime} ,A_{c} ,B_{c} ,C_{c} ,D_{c} ,A_{c}^{\prime} ,B_{c}^{\prime} ,C_{c}^{\prime} ,D_{c}^{\prime}$ are transformed through the corresponding pose matrix.
- to obtain a new keel side guard plate surrounding space $A_{t0} B_{t0} C_{t0} D_{t0} - A_{t0}^{\prime} B_{t0}^{\prime} C_{t0}^{\prime} D_{t0}^{\prime}$;
- Determine whether the line $A_{t0} A_{t0}^{\prime} ,D_{t0} D_{t0}^{\prime}$ is between surface $A_{c0} B_{c0} A_{c0}^{\prime} B_{c0}^{\prime}$ and $C_{c0} D_{c0} C_{c0}^{\prime} D_{c0}^{\prime}$. The judging conditions are :
  10 $$Z_{\Delta } \le Z_{\square } \le Z_{\nabla }$$

In the formula :$Z_{\Delta }$ represents the Z coordinate of any point on surface $C_{c0} D_{c0} C_{c0}^{\prime} D_{c0}^{\prime}$.$Z_{\square }$ represents the Z coordinate of any point on line $A_{t0} A_{t0}^{\prime} ,D_{t0} D_{t0}^{\prime}$.$Z_{\nabla }$ represents the Z coordinate of any point on surface $A_{c0} B_{c0} A_{c0}^{\prime} B_{c0}^{\prime}$.

The collision between the upper guard plate of the keel of the digging anchor digging machine and the drill bit of the water drilling rig is mathematically expressed as : The collision between the edge $A_{t} D_{t}$ of the bit bounding box $A_{t} B_{t} C_{t} D_{t} - A_{t}^{\prime} B_{t}^{\prime} C_{t}^{\prime} D_{t}^{\prime}$ and the plane $A_{s} B_{s} C_{s} D_{s}$ or the collision between the upper guard plate boundary *A*_*s*_*D*_*s*_ and the bit bounding box surface $A_{t} B_{t} C_{t} D_{t}$. Considering that plane $A_{t} B_{t} C_{t} D_{t}$ and plane $A_{s} B_{s} C_{s} D_{s}$ are always perpendicular to the *X*_0_*O*_0_*Y*_0_ plane of the world coordinate when the rig and the keel are adjusted, the collision detection can be projected onto the *X*_0_*O*_0_*Y*_0_ plane.

**Fig. 9:**
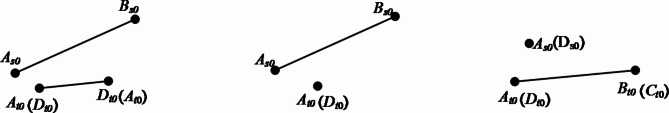
Projection situation.

Projecting the bounding box and plane boundary onto the *X*_0_*O*_0_*Y*_0_ plane yields several cases as shown in Fig. 9. From left to right, the first two cases are mathematically expressed as the projection of the collision between the edge $A_{t} D_{t}$ of the bit bounding box $A_{t} B_{t} C_{t} D_{t} - A_{t}^{\prime} B_{t}^{\prime} C_{t}^{\prime} D_{t}^{\prime}$ and the plane $A_{s} B_{s} C_{s} D_{s}$, and the third is the collision between the upper guard plate boundary *A*_*s*_*D*_*s*_ and the bit bounding box surface $A_{t} B_{t} C_{t} D_{t}$. The second case is the case when *A*_*t*_ and *D*_*t*_ are in the same *Z* coordinate on the world coordinate.

According to the analysis of actual working conditions, for the first two cases in Fig. 9, only when the point *A*_*t*0_ (*D*_*t*0_) does not pass and is located below the straight line *A*_*s*0_-*B*_*s*0_, no collision occurs. For the third case in Fig. 9, no collision occurs only when the point *A*_*s*0_(*D*_*s*0_) does not pass and is located above the line *A*_*z*0_ (*D*_*z*0_) -*B*_*z*0_ (*C*_*z*0_). Therefore, the judging condition of the collision between drill bit and keel upper guard plate is :

11 $$\left\{ \begin{gathered} \begin{array}{*{20}c} {\left[ {\begin{array}{*{20}c} X \\ {Y_{0} } \\ \end{array} } \right] = (1 - t)A + tB} & {t \in [0,1]} \\ \end{array} \hfill \\ \hfill \\ \begin{array}{*{20}c} {Y_{0}> Y} & {x_{{A_{t0} (D_{t0} )}}> x_{{A_{s0} (D_{s0} )}} } \\ \end{array} \hfill \\ \begin{array}{*{20}c} {Y_{0} < Y} & {x_{{A_{t0} (D_{t0} )}} \le x_{{A_{s0} (D_{s0} )}} } \\ \end{array} \hfill \\ \end{gathered} \right.$$

In the formula : *t* unknown parameters ; *A* and *B* are the endpoint coordinates of the projection line segment of the collision surface on the *X*_0_*O*_0_*Y*_0_ surface ; *X* and *Y* are the horizontal and vertical coordinates of the points to be detected.

The collision between the keel support cylinder and the drilling rig support cylinder is mathematically expressed as : The collision between cylinder $A_{Z1} B_{z1}$ and cylinder $A_{lc} B_{lc}$ and the collision between cylinder $A_{z1}^{\prime} B_{z1}^{\prime}$ and cylinder $A_{lc}^{\prime} B_{lc}^{\prime}$. Considering that when the two cylinders do not collide, the nearest point distance between the straight lines of the central axes of the two cylinders must be greater than the radius sum of the two cylinders. Therefore, the collision judgment condition between the keel support cylinder and the drilling rig support cylinder is :

$$R_{lc} + r_{z} \le d_{zlc}$$

In the formula : *R*_*lc*_ is the diameter of the keel support cylinder ; *r*_*z*_ is the diameter of drilling rig support cylinder ; *d*_*zlc*_ is the nearest point distance between the straight lines where the central axes of the two cylinders are located, and its calculation formula is shown in Eq. (12).

12 $$\left\{ \begin{gathered} \begin{array}{*{20}c} {\overrightarrow {{d_{1} }} = B_{z1} - A_{z1} } & {\overrightarrow {{d_{2} }} = B_{lc} - A_{lc} } \\ \end{array} \hfill \\ \overrightarrow {r} = A_{z1} - A_{lc} \hfill \\ \begin{array}{*{20}c} {L_{1} (s) = A_{z1} + s\overrightarrow {{d_{1} }} } & {L_{2} (t) = A_{lc} + t\overrightarrow {{d_{2} }} } \\ \end{array} \hfill \\ \hfill \\ \left[ {\begin{array}{*{20}c} {\overrightarrow {{d_{1} }} \cdot \overrightarrow {{d_{1} }} } & { - \overrightarrow {{d_{1} }} \cdot \overrightarrow {{d_{2} }} } \\ {\overrightarrow {{d_{1} }} \cdot \overrightarrow {{d_{2} }} } & { - \overrightarrow {{d_{2} }} \cdot \overrightarrow {{d_{2} }} } \\ \end{array} } \right]\left[ {\begin{array}{*{20}c} s \\ t \\ \end{array} } \right] = \left[ {\begin{array}{*{20}c} { - \overrightarrow {{d_{1} }} \cdot \overrightarrow {r} } \\ { - \overrightarrow {{d_{2} }} \cdot \overrightarrow {r} } \\ \end{array} } \right] \hfill \\ d_{zlc} = \sqrt {(s_{x} - t_{x} )^{2} + (s_{y} - t_{y} )^{2} + (s_{z} - t_{z} )^{2} } \hfill \\ \end{gathered} \right.$$

In the formula : *L*_1_ and *L*_2_ are the parameter equations of the straight lines of the central axes of the two cylinders ; *s*, *t* are the nearest points on two lines.

Considering the collision between the drill pipe and the cutting part of the anchor digging machine and the collision between the drill pipe holder and the keel support cylinder is also simplified as the collision of two cylinders. In the principle of collision detection, it is consistent with the collision detection principle between the keel support cylinder and the drilling rig support cylinder. This article is not repeated.

The collision between the drill pipe holder and the guard plate on the keel is mathematically expressed as the collision between the cylinder *A*_*j*_*B*_*j*_ and the surface *A*_*s*_*B*_*s*_*C*_*s*_*D*_*s*_. By analyzing the relationship between the two, it can be seen that the minimum distance between the line segment *A*_*j*_*B*_*j*_ and the surface *A*_*s*_*B*_*s*_*C*_*s*_*D*_*s*_ is always greater than the radius of the cylinder *A*_*j*_*B*_*j*_ when the drill pipe holder and the upper guard plate of the keel collide. Combined with the (5) plane equation, the collision detection conditions of drill pipe gripper and keel upper guard plate are obtained :

$$r_{j} < d_{js}$$

In the formula : *r*_*j*_ is the radius of the bounding box of the drill pipe holder. *d*_*js*_ is the minimum distance between the line segment *A*_*j*_*B*_*j*_ and the surface *A*_*s*_*B*_*s*_*C*_*s*_*D*_*s*_. Its size can be calculated by Formula ( 13 ).

13 $$\left\{ \begin{gathered} \begin{array}{*{20}c} {t_{1} = \frac{{\overrightarrow {{n_{s} }} }}{{\left| {\overrightarrow {{n_{s} }} } \right|}}(A_{j} - P_{0} )} & {t_{2} = \frac{{\overrightarrow {{n_{s} }} }}{{\left| {\overrightarrow {{n_{s} }} } \right|}}(B_{j} - P_{0} )} \\ \end{array} \hfill \\ d_{js} = \left\{ {\begin{array}{*{20}c} {\min (t_{1} ,t_{2} )} & {t_{1} t_{2}> 0} \\ 0 & {t_{1} t_{2} < 0} \\ \end{array} } \right. \hfill \\ \end{gathered} \right.$$

In the formula :$\overrightarrow {{n_{s} }}$ is the normal vector of planar *A*_*s*_*B*_*s*_*C*_*s*_*D*_*s*_.$P_{0}$ is any known point on the planar *A*_*s*_*B*_*s*_*C*_*s*_*D*_*s*_. *t*_1_*t*_2_ is the possible nearest distance between line *A*_*j*_*B*_*j*_ and surface *A*_*s*_*B*_*s*_*C*_*s*_*D*_*s*_. *d*_*js*_ is the nearest distance between point and face.

## Simulation analysis

Based on the kinematic analysis of the equipment, the AABB bounding box is used to mathematically model the parts where the equipment may collide, and the basic principle of system collision detection is described. The following MB670 type anchor digging machine and self-developed airborne water drilling rig as the research object, and based on the basic size of the equipment in MATLAB simulation analysis. The basic parameters of the device in the non-running state are ( length default unit mm ) :* α* = 5.7°, *θ*_1_ = 5.7°, *θ*_2_ = −5.7°, *θ*_4_ = 0°, a_1_ = 2310, a_2_ = −404, a_4_ = −304.5, d_3_ = 104, d_5_ = −405.5, *d*_*oc*_ = 3200, *h*_*c*_ = 2300, *r*_*cx*_ = 1470, *r*_*cy*_ = 182.73 , *r*_*cz*_ = 614, *d*_*os*_ = 5100, *h*_*os*_ = 400, *r*_*sx*_ = 1090, *r*_*sz*_ = 927.5, *d*_*xz1*_ = 210, *d*_*zy1*_ = 208, *d*_*zz1*_ = 125, *l*_*z*_ = 1250, *r*_*z*_ = 92, *d*_*xj*_ = 1527.6, *d*_*zj*_ = 405.5, *l*_*j*_ = 386.8, , *r*_*j1*_ = 145, *r*_*j2*_ = 26.5, *r*_*tx*_ = 370, *r*_*ty*_ = 200, *r*_*tz*_ = 177.5, *d*_*og*_ = 2560, *h*_*og*_ = 7250, *l*_*g*_ = 1070, *r*_*g*_ = 580, *r*_*xalc*_ = 6030, *r*_*yalc*_ = 310, *r*_*zalc*_ = 737.5, *r*_*xblc*_ = 5560, *r*_*yblc*_ = 944, *r*_*zblc*_ = 737.5 .

**Fig. 10:**
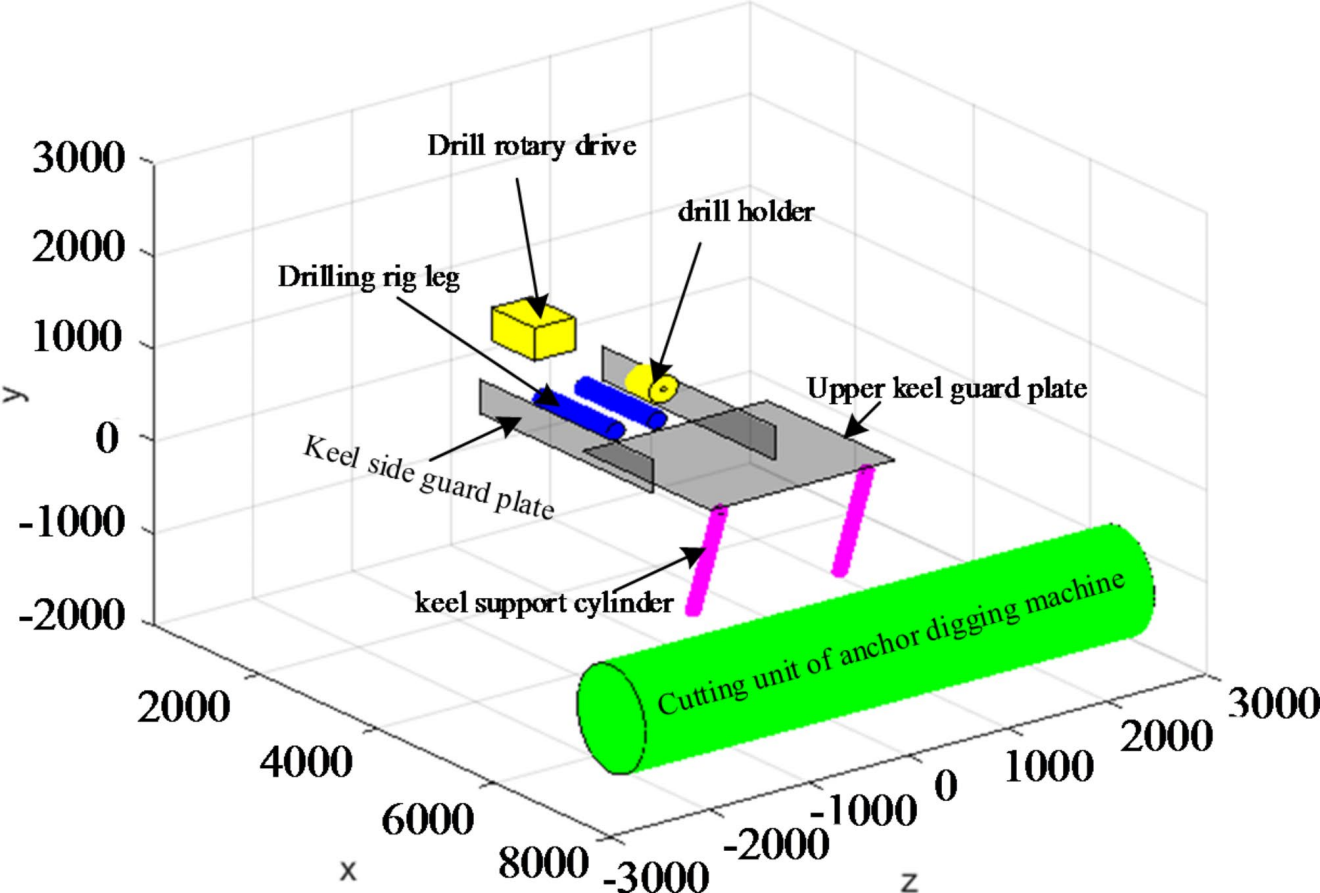
Initial state of equipment.

Based on the above parameter setting and the mathematical model established in the second section, the bounding box of key components at the initial position of the equipment is obtained by MATLAB modeling, as shown in Fig. 10.

### Fixed-point drilling test

**Fig. 11:**
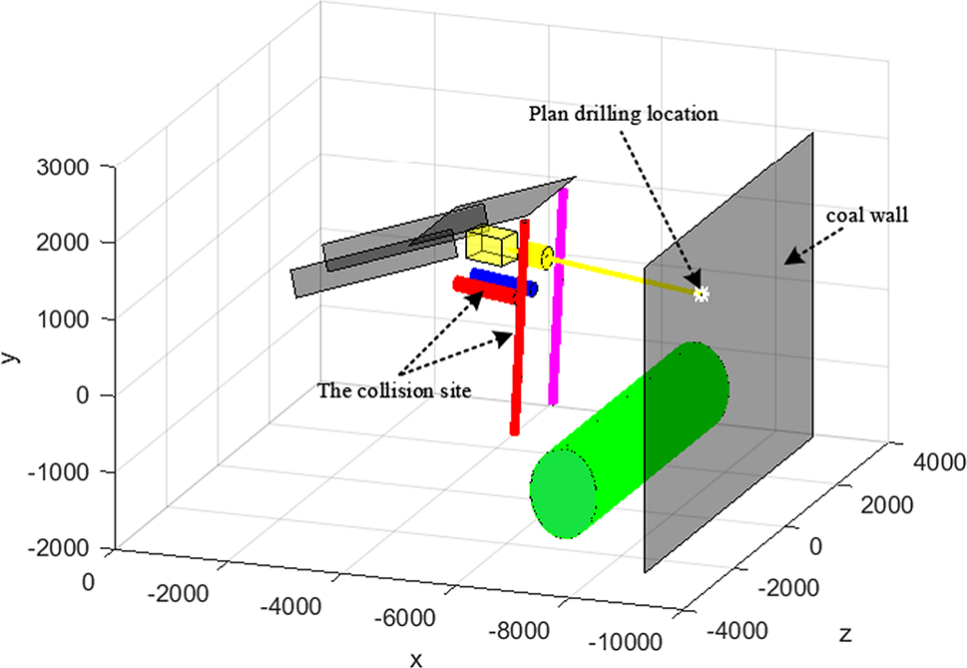
Pre-working position.

**Fig. 12:**
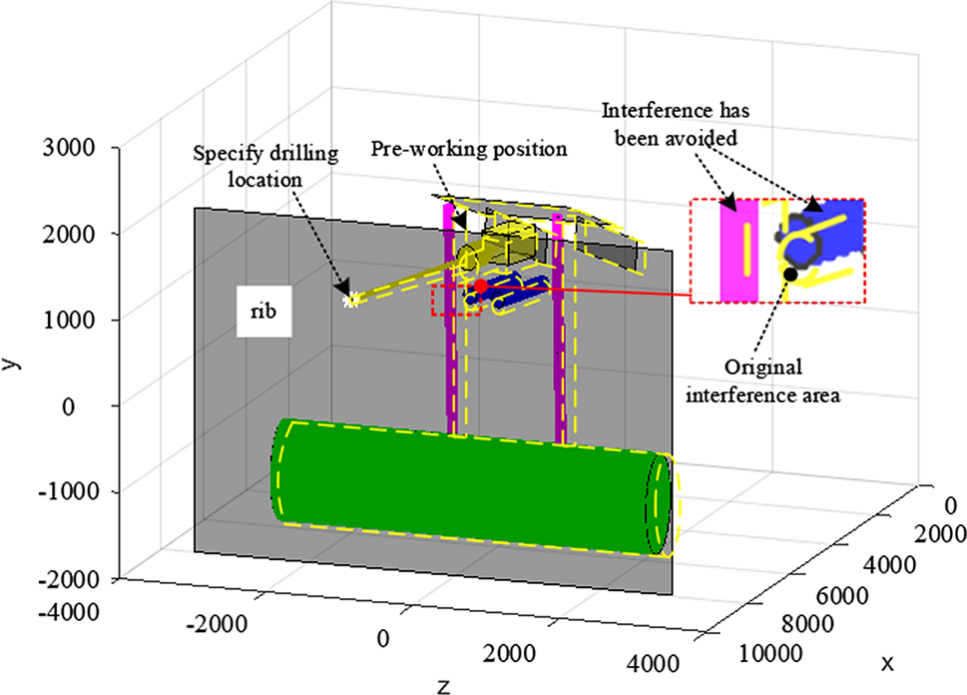
working position.

In order to verify the feasibility of the study, now fixed-point drilling collision detection verification. Water drilling rig installed in the 4-m long drill pipe, pre-determined on the current position of the equipment under the world coordinates P ( 9000,1326,1330 ) for drilling. The joint variables obtained by inverse motion are :* θ*_1_ = 20.9°, *θ*_2_ = −20.8°, *θ*_4_ = 15.3°, a_2_ = −2016, a_4_ = 760.5 . Through simulation analysis, the pre-working position of the equipment is shown in Fig. 11:

As can be seen from Fig. 11, the bounding box of the rig support cylinder and the keel support cylinder is shown in red, which indicates that the boring machine 's drilling operation at the current position ( 9000,1326,1330 ) will cause the right support cylinder of the keel to collide with the right support cylinder of the water exploration rig. It indicates that the roadheader cannot drill the specified point at the current position. Figure 11 shows that the main reason for the interference of the equipment is that the equipment rotates too far to the right and the target point is too far away from the anchor machine. In other words, in order to avoid interference roadheader should run to the right side to adjust the world coordinate position. The tunneling is moved to the right for 200, and the inverse motion is solved to obtain a new joint variable, and the variable is substituted into the simulation program to obtain the working position of the drilling rig as shown in Fig. 12.

From Fig. 12, it can be seen that the bounding box of the drilling rig support cylinder and the keel support cylinder shows that the standard red has been removed, the roadheader has been properly adjusted, and the fixed-point drilling of the water drilling rig does not cause interference problems and can be drilled.

### Collision test

**Fig. 13:**
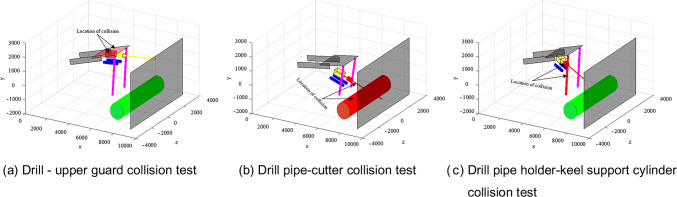
collision test.

The above simple examples verify the feasibility of collision detection method for fixed-point drilling. Considering that there are many parts where collision occurs during equipment operation, it is no longer described one by one. This section directly tests the feasibility of the collision detection method in MATLAB by giving specific joint variables. Through the test, it is known that the two equipments involved in this paper only have the collision possibility between the drill bit and the upper guard plate, the drill pipe and the keel support cylinder, the drill pipe and the cutting part of the roadheader, the keel support cylinder and the drilling rig support cylinder during the operation( Specific readers can substitute the parameters listed in this article to verify themselves ). Therefore, this section only shows the collision test results between the drill bit and the upper guard plate, the drill pipe and the keel support cylinder, the drill pipe and the cutting part of the roadheader. The final result is shown in Fig. 13.

It can be seen from Fig. 13 (a) that the bounding box of the drill bit and the upper guard plate is shown in red, indicating that the collision detection of the drill bit upper guard plate is successful . According to Fig. 13 (b), the bounding box of the drill pipe and the cutting part of the roadheader is shown in red, indicating that the collision detection of the guard plate on the drill bit is successful . In Fig. 13 (c), the box of drill pipe and roadheader keel support cylinder is shown as red, indicating that the collision detection of the guard plate on the drill bit is successful.To sum up, it can be concluded that the theory in Sections "system mathematical model" and "Collision detection method" of this paper can accurately verify the feasibility of the equipment before operation.

At the same time, the recording of the simulation test results shows that the horizontal rotation angle range of the exploration and drainage drilling rig is − 15.17° ~ 15.17°, and the adjustment range of the pitch angle is − 10.03° ~ 5.14°. In the future, the no-load test of the airborne water drilling rig will be carried out to verify whether the simulation results match the actual situation.

## Field test

### Experimentation

In order to verify that the collision detection method proposed in this paper can be applied to practice. It is ensured that the designed collision detection system can assist the efficient advanced drilling operation in coal mine and reduce the labor intensity of workers. The industrial test of water drilling rig and key technology of anchor digging machine was carried out in 51109 main transport channel of Lijiahao Coal Mine of Guoneng Baotou Energy Co. , Ltd. The 51109 main transport crossheading is opened in the 51109 auxiliary withdrawal channel in the underground. Driving east at 90°35′15"azimuth. The main transportation gateway of No.51109 is used for air intake and main transportation during the mining of No.51109 working face. The main roof of the excavation section is fine and medium grained sandstone, the lithology of the direct roof is sandy mudstone, the lithology of the direct bottom is sandy mudstone and mudstone, and the lithology of the old bottom is siltstone and sandy mudstone.

**Fig. 14:**
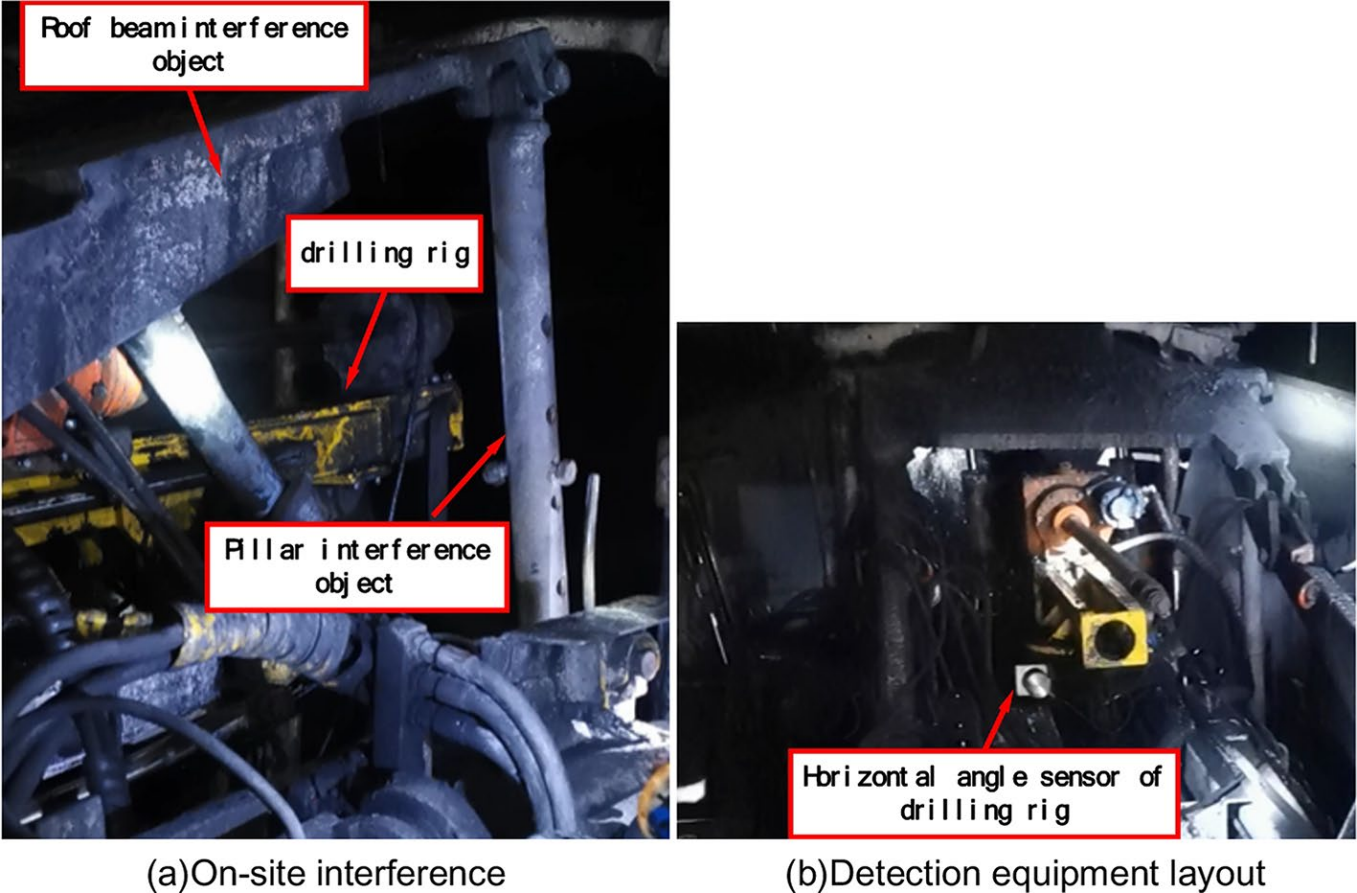
On-site installation of water drilling rig of anchor digging machine.

The anchor digging machine and the water drilling rig are installed on the site, and the field interference of the simulation conditions is shown in Fig. 14(a). The layout of the detection equipment is shown in Fig. 14(b).

The main test contents:

1. By comparing the simulation results, the adjustment range of drilling parameters of the water drilling rig and the pitch interference characteristics of the water drilling rig at different positions are verified.
2. The reliability of the drilling collision detection system of the water drilling rig is verified.

The test steps are as follows:

1. The main machine and operating table of the onboard water drilling rig of the anchor digging machine are fixedly installed on the anchor digging machine. Install the pumping station and electric control box of the on-board water drilling rig of the anchor digging machine on the No.1 vehicle.
2. The hydraulic main pipeline and cable of the water drilling rig are laid along the cable groove of the anchor digging machine.
3. Start the system, adjust the attitude angle of the water drilling rig under no-load conditions, record the angle and position of the water drilling rig that can realize the drilling, test whether the adjustment range of the pose parameters of the water drilling rig matches the simulation results, and detect the pitch interference characteristics of the water drilling rig at different positions.
4. The drilling position and drilling angle are selected, and the collision detection system is used to judge whether the current position of the anchor digging machine can realize the drilling operation without interference. If it is judged that no collision occurs, the drill pipe is directly installed to carry out the water exploration and drainage operation.
5. If the system determines that the current position of the anchor digging machine and the water drilling rig will collide, re-adjust the position of the anchor digging machine, and repeat the fourth step until the collision system determines that the specified position will not collide with the specified angle drilling operation.
6. After the test of water exploration and drainage operation is completed, the executive components of the main machine of the water drilling rig are operated to the initial state to ensure that the normal operation of the anchor digging machine is not disturbed. Shut down the pumping station, cut off the power

**Fig. 15:**
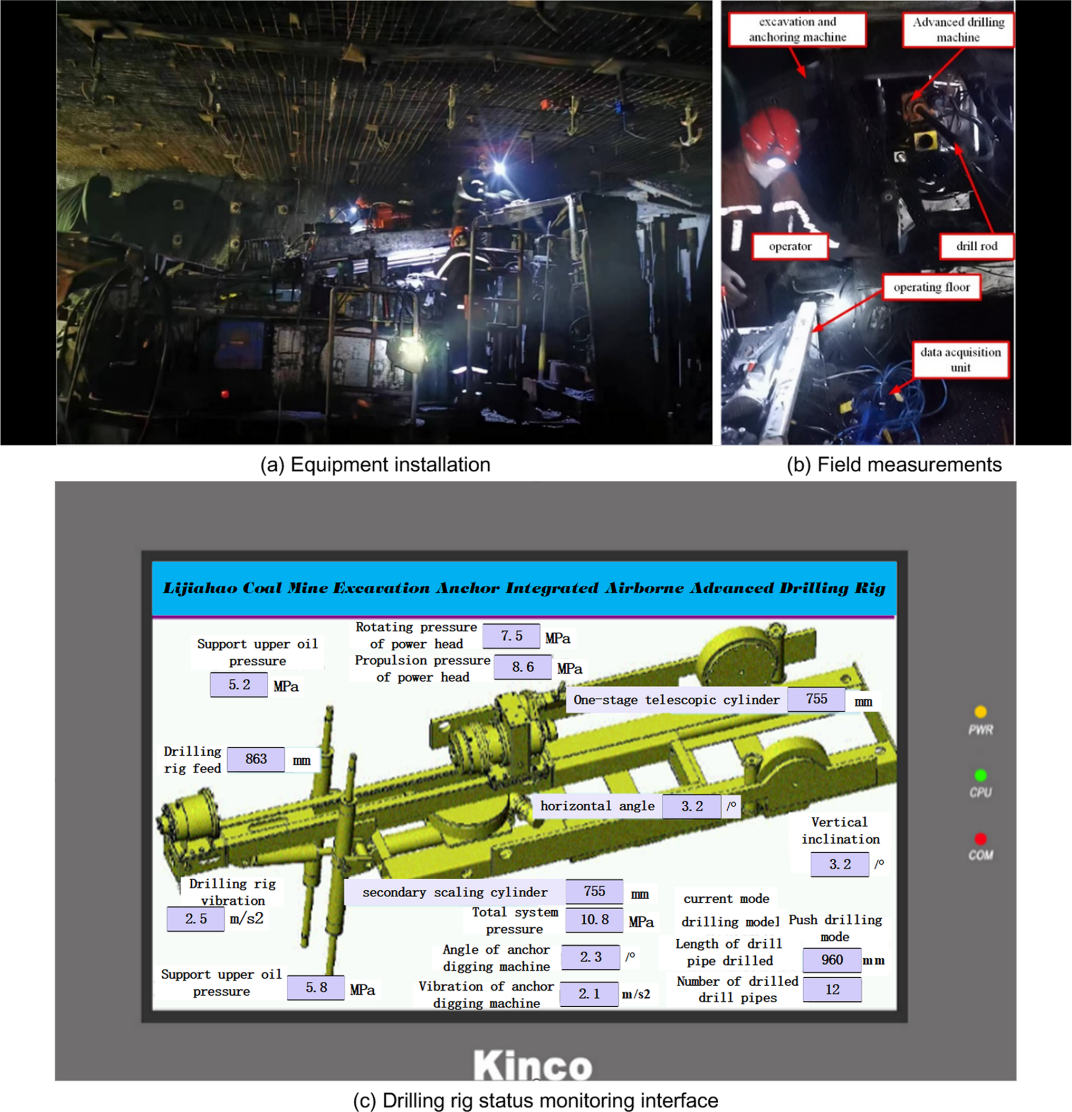
Installation and test of water drilling rig of anchor digging machine on site.

The field test of the anchor digging machine and the water drilling rig is shown in Fig. 15.

The monitoring interface of the on-board water drilling rig of the anchor digging machine is shown in Fig. 15 (c).Through the man–machine interface storage function, the equipment status value and drilling depth data of the water drilling rig are recorded and uploaded in real time.

### Experimental result

**Fig. 16:**
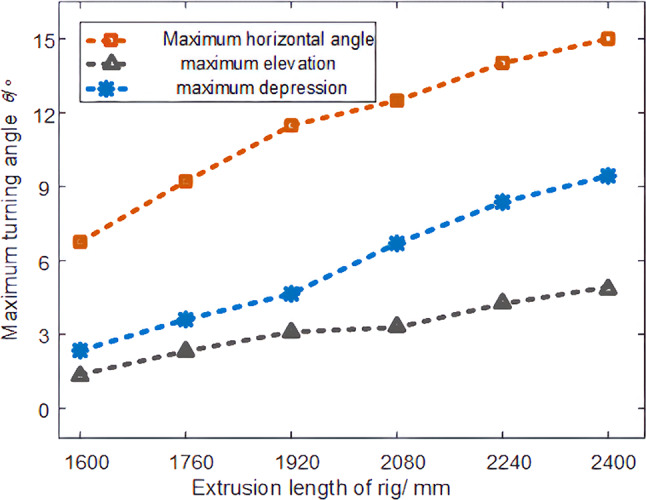
Record of drilling rig rotation angle.

Through the field test, the adjustment range of the extension length of the water drilling rig is shown in Fig. 16.

The following results are obtained by analyzing Fig. 16 and Table 2:

1. The azimuth adjustment range of the water drilling rig is: ± 15°. The angle is mainly restricted by the position of the advance support column of the anchor digging machine, and the no-load test results are basically consistent with the simulation results.
2. The adjustment range of the pitch angle of the water drilling rig is:−10° ~ 5°(+ is the upward pitch angle, -is the downward pitch angle ) The angle adjustment is mainly restricted by the advance support plate of the anchor digging machine, and the no-load test results are basically consistent with the simulation results.

**Table 2:** Key point coordinates.

Extrusion length of rig /mm	Maximum horizontal angle θ/°	Maximum elevation θ/°	Maximum depression θ/°
1600	6.8	1.2	2.3
1760	9.1	2.3	3.5
1920	11.4	3.0	4.6
2080	12.6	3.3	6.7
2240	13.5	4.1	8.3
2400	15	4.7	9.8

**Fig. 17:**
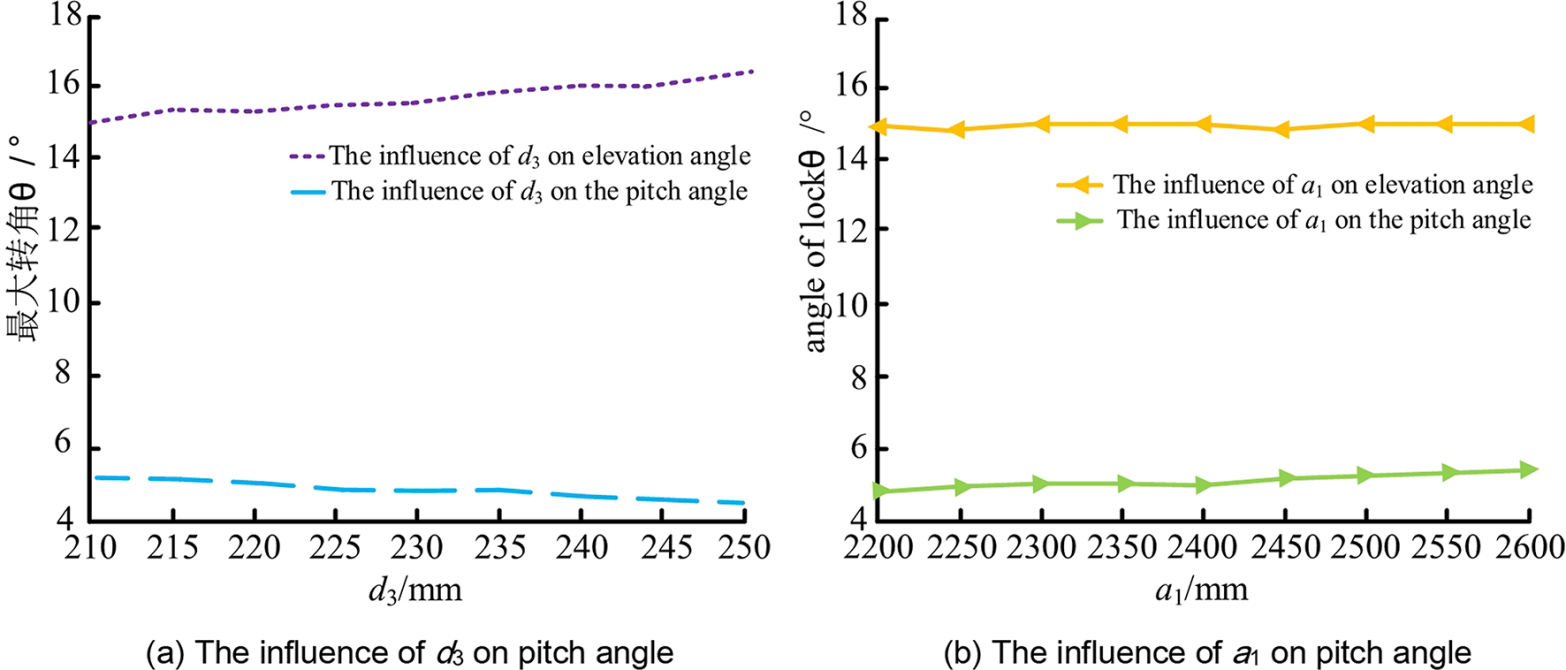
Rig Angle Record.

Through the field test, the installation position and pitch parameter adjustment range of the water drilling rig are shown in Fig. 17.

The installation position of the water drilling rig on the airborne drilling rig is mainly determined by the height difference between the size drilling rig and the winch ear, and the length of the keel connecting the winch ear to the tail of the keel. The figures are represented by *d*_3_ and *a*_1_ respectively. In order to analyze the influence of the installation position of the water drilling rig on the pitch interference characteristics, the pitch interference characteristics at different installation positions of the airborne drilling rig are obtained through field implementation.

It can be seen from Fig. 17 that the influence of *a*_1_ change on the pitch angle is very small, the influence on the elevation angle is almost 0, and the influence on the pitch angle is slightly larger. The change of *d*_3_ has a great influence on the pitch angle and elevation angle, which indicates that the elevation angle range can be adjusted by changing *d*_3_. If you want to change the elevation angle, you can give priority to changing *d*_3_ ; if you want to change the pitch angle, you can give priority to changing *d*_3_ and then consider *a*_1_.

In the test, the drilling operation is carried out by deliberately specifying the position where the initial attitude of the anchor digging machine cannot be directly drilled. In order to test whether the collision detection system can correctly judge the working condition. Through multiple rounds of adjustment and testing, it is finally determined that the collision detection system can be used to guide the attitude adjustment of the equipment, and can perform real-time interference characteristic analysis and collision detection on the equipment.

## Conclusion

This paper gives a brief introduction to the working conditions of the anchor digging machine airborne water drilling rig. Based on the kinematics analysis of the equipment, the kinematics model of the equipment is established. At the same time, combined with the bounding box collision detection technology, the key components of the equipment are mathematically modeled, and the corresponding collision detection method is designed.

Firstly, based on the collision detection method proposed in this paper, the MB670 anchor digging machine and the self-developed airborne water drilling rig are taken as the research objects. The simulation analysis is carried out by MATLAB. The simulation shows that the collision detection method can be used to guide the attitude adjustment of the equipment during the drilling operation and can realize the interference characteristic analysis and collision detection between the equipment. It provides a theoretical basis for the research of automatic control theory of equipment automatic tunneling and drilling.

By recording the angle and pose of the airborne water drilling rig under no-load condition, the drilling parameter adjustment range of the water drilling rig is obtained. The test results are basically consistent with the simulation results. There is an error of less than 0.2 between the results of no-load operation and simulation analysis. The main reason is that the no-load test results are all measured manually and the collision between the equipment is judged manually. This error cannot be avoided and is within a reasonable range. Finally, the feasibility and reliability of the collision detection system are verified based on several rounds of industrial experiments.

## Electronic supplementary material

Below is the link to the electronic supplementary material.

Supplementary Material 1

## Data Availability

All data generated or analysed during this study are included in this published article [and its supplementary information files].
